# Elucidating Interactions and Conductivity of Newly Synthesised Low Bandgap Polymer with Protic and Aprotic Ionic Liquids

**DOI:** 10.1371/journal.pone.0068970

**Published:** 2013-07-09

**Authors:** Pankaj Attri, Seung-Hyun Lee, Sun Woo Hwang, Joong I. L. Kim, Sang Woo Lee, Gi-Chung Kwon, Eun Ha Choi, In Tae Kim

**Affiliations:** 1 Plasma Bioscience Research Center, Department of Electrical and Biological Physics, Kwangwoon University, Seoul, Korea; 2 Graduate School of Information Contents, Kwangwoon University, Seoul, Korea; 3 Department of Chemistry, Kwangwoon University, Seoul, Korea; University of Calgary, Canada

## Abstract

In this paper, we have examined the conductivity and interaction studies of ammonium and imidazolium based ionic liquids (ILs) with the newly synthesised low bandgap polymer (Poly(2-heptadecyl-4-vinylthieno[3,4-d]thiazole) (PHVTT)). Use of low bandgap polymers is the most suitable way to harvest a broader spectrum of solar radiations for solar cells. But, still there is lack of most efficient low bandgap polymer. In order to solve this problem, we have synthesised a new low bandgap polymer and investigated its interaction with the ILs to enhance its conductivity. ILs may undergo almost unlimited structural variations; these structural variations have attracted extensive attention in polymer studies. The aim of present work is to illustrate the state of art progress of implementing the interaction of ILs (protic and aprotic ILs) with newly synthesised low bandgap polymer. In addition to this, our UV-Vis spectroscopy, confocal Raman spectroscopy and FT-IR spectroscopy results have revealed that all studied ILs (tributylmethylammonium methyl sulfate ([N_1444_][MeSO_4_] from ammonium family) and 1-methylimidazolium chloride ([Mim]Cl, and 1-butyl-3-methylimidazolium chloride ([Bmim]Cl from imidazolium family**)** have potential to interact with polymer. Our semi empirical calculation with help of Hyperchem 7 shows that protic IL ([Mim]Cl) interacts strongly with the low bandgap polymer through the H-bonding. Further, protic ILs shows enhanced conductivity than aprotic ILs in association with low bandgap polymer. This study provides the combined effect of low bandgap polymer and ILs that may generate many theoretical and experimental opportunities.

## Introduction

The blend of polymers and ionic liquids (ILs) has charmed the modern research, [1]–[10] specifically as replacements for current solid-state polyelectrolytes in energy devices, such as dye-sensitized solar cells, [11] supercapacitors, [12] lithium ion batteries, [13] and fuel cells [14]. An IL completely consists of the weak coordination of ions such as an organic cation and the inorganic or organic anion [15]–[18]. The potential utilization and applications of ILs have been rapidly increased in all scientific fields by several researchers [19]–[27]. Apparently, the physicochemical properties of ILs are quite sensitive toward the structure and nature of cations and anions [14], [15], [17]. Their resulting unique physiochemical properties (e.g., negligible vapor pressure, high conductivity, wide electrochemical window, high chemical and thermal stability) have motivated, in large part, this will help in investigating the properties and uses of polymer/IL mixtures. There are many polymers that have been explored in IL mixtures to increase their conductivity such as homopolymers, [28] copolymers, [29] crosslinked polymers, [30] and block copolymers [31]–[36]. It has been reported that the crosslinked ionic gels of poly(methyl methacrylate) (PMMA) and 1-ethyl-3-methylimidazolium bis(trifluoromethylsulfonyl)imide (EMIm-TFSI) has high ionic conductivities even at 100°C [30]. Similarly, Gwee et al., [37] showed that mixtures of a block copolymer, poly(styrene-b-methyl methacrylate) (SbMMA), and 1-ethyl-3-methylimidazolium bis (trifluoromethylsulfonyl)imide (EMIm-TFSI) IL, has significant impact on the both morphology and microdomain orientation that leads to increase in high ionic conductivity and also this system has enhanced conductivity as compared to homopolymer/IL mixture. Further, Cheng et. al., [38] showed that addition of 1-butyl-4-methylpyridinium bis(trifluoromethanesulfonyl)imide (BMPyTFSI) to the P(EO)_20_LiTFSI electrolyte results in an increase in the ionic conductivity, and at high BMPyTFSI concentration (BMPy+/Li+ = 1.0), the ionic conductivity reaches the value of 6.9×10^−4^ S/cm at 40°C. Thomas E. Suttoz, [39] revealed that Poly(ethylene oxide), PEO, or poly(vinylidenefluoride-*co*-hexafluoropropene), PVdF-HFP, and 1-*n*-propyl-2,3-dimethylimidazolium tetrafluoroborate (MMPIBF_4_) and 1-*n*-propyl-2,3-dimethylimidazolium hexafluorophosphate (MMPIPF_6_) ILs exhibited the enhanced ionic conductivity. Whereas, Döbbelin et al., [40] demonstrated that ILs showed very good performance as permanent conductivity enhancers in PEDOT: PSS films. But, still there is lack of research on the interaction and conductivity of ILs with low bandgap polymers.

Low bandgap polymers have attracted much attention due to the broad range of small and large area applications in organic photovoltaic devices (OPVs) [41]. Extensive researchin the field of OPVs have led to increase in the power conversion efficiencies over past decade. There are many reasons for the interest in OPVs such as low cost, low thermal budget, solution processing, flexible substrates and a very high speed of processing. In the early 1990s, the OPVs showed low efficiencies and short lifetimes [42]. While in the recent years, both the efficiency and lifetime of OPVs have changed dramatically [43], [44]. There are several factors that influence the efficiency of OPVs, among them one of the most important factors is structure of the polymer [45]. During the last decade several research groups have reported the synthesis and use of low bandgap polymers in OPVs [46]–[49]. In low-bandgap polymers, electron donating and accepting moieties are copolymerized and form the repeat unit of the polymeric chain. The two moieties are conjugated and form a ladder of energy levels typically exhibiting a smaller energy gap between the frontier orbitals, highest occupied molecular orbital (HOMO) and lowest unoccupied molecular orbital (LUMO), than the isolated uncoupled donor and acceptor [50]. However, most of these materials suffer from the inherent disadvantages of either lacking a broad absorption range (band gap [Eg] ≈1.6–2.0 eV), which limits the usage of the full solar spectrum, or having a relatively low carrier mobility [51]–[55].

In order to solve the above discussed problem, we have synthesized a new low bandgap polymer using stille reaction. In addition to this, we have also revealed the interaction of this low band gap polymer with ammonium and imidazolium family ILs and exposed that there is an increase in conductivity of polymer when interacted with ILs.

## Materials and Methods

### Materials

The reagents used for the synthesis of polymer and ILs were supplied by Aldrich Chemical Co. (USA). All chemicals and reagents were used without any further purification. 1-methyl imidazolium chloride ([Mim]Cl of high purity, obtained from Sigma–Aldrich (Yongin, Korea), were used without further purification. Rest of ILs were synthesized in laboratory and analysed using ^1^H-NMR, the preparation is given below.

### Synthesis of ILs

#### Synthesis of 1-Butyl-3-methylimidazolium chloride ([Bmim]Cl

To a vigorously stirred solution of 1-methylimidazole (1.25 mol) in toluene (125 mL) at 0°C, 1-chlorobutane (1.38 mol) was added. The solution was heated to reflux at 110°C for 24 h, after which it was placed in a freezer at −20°C for 12 h. The toluene was decanted and the remaining viscous oil/semi-solid was recrystallized from acetonitrile and then repeatedly recrystallized from ethyl acetate to yield a white crystalline solid, which was further dried under reduced pressure to give [Bmim]Cl in approximately 86% yield. ^1^H-NMR (400 MHz, DMSO-*d*_6_): δ = 10.54 (1H, s), 7.55 (1H, m), 7.40 (1H, m), 4.26 (2H, t, *J = *7.3 Hz), 4.11 (3H, s), 1.82 (2H, m), 1.30 (2H, m), 0.89 (3H, t, *J = *7.3 Hz).

#### Synthesis of tributylmethylammonium methyl sulfate [N_1444_][MeSO_4_]

Dissolve the dimethyl sulfate (0.1 mol) in anhydrous toluene (50 mL) followed by slowly dropwise addition of a solution of tributylamine (0.2 mol) in anhydrous toluene (150 mL). The mixture was continuously cooled in an ice-bath under nitrogen with care being taken to maintain the reaction temperature below 25°C. After complete addition of the dimethyl sulfate, the reaction mixture was then stirred at room temperature for 6 h. The IL phase was washed with ethyl acetate (3×15 mL). After washing, the residual water was removed by heating under reduced pressure. [N_1444_][MeSO4] was obtained with approximately 89% yield.^ 1^H-NMR (400 MHz, DMSO-*d*_6_): δ = 3.36 (s, 3H), 3.20 (m, 6H), 2.95 (s, 3H), 1.60 (m, 6H), 1.30 (6H, q, *J* = 7.5 Hz), 0.92 (9H, t, *J* = 7.3 Hz).

#### Synthesis of polymer using stille reaction Poly(2-heptadecyl-4-vinylthieno[3,4-d]thiazole) (PHVTT)

4,6-dibromo-2-heptadecyl-thieno[3,4-d]thiazole [46] (0.35 g, 0.650 mmol), 1-(dibutyl((E)-2-(tributylstannyl)vinylstannyl)butane (0.39 g, 0.065 mmol), and Pd(PPh_3_)_4_(0) (0.02 g, 0.025 mmol) were placed in 100-mL 3-neck round bottom flask equipped with a condenser, purged with N_2_ gas and subsequently dissolved in 20 mL of toluene, from which oxygen was removed by purging with nitrogen for 1 h. The resulting mixture were stirred at 120°C for 72 hours, after which 20 µL of 2-bromothiophene was injected as a capping agent. The reaction was stirred for 2 hr at 120°C before 20 µL of 2-(tributyltin) thiophene was injected to complete the end-capping. The reaction mixtures were cooled and filtered to separate a blue solid as polymer. The blue polymer as product was purified by ethanol and acetone to yield a pure conducting a deep blue solid. The yield was 81%. It was soluble in common organic solvents such as CHCl_3_, CH_2_Cl_2_, and THF. Mn = 9980, Mw = 26499, PD = 2.655. ^1^H NMR (400 MHz, CDCl_3_): δ = 7.19(m, 1H), 7.17(m, 1H), 2.92(m, 2H), 1.79(m, 2H), 1.22(m, 28H), 0.81(m, 3H) ppm; ^13^C NMR (400 MHz, CDCl_3_): δ = 22.71, 29.40, 29.79, 31.95, 76.68, 77.00, 77.32, 133.01, 140.97, 142.91, 170.90 ppm; FT-IR (KBr) ν 3002, 2941,2880,1620 cm^−1^.

#### Measurements

The synthesized compounds were identified by ^1^H NMR and ^13^C NMR spectra that were obtained using a Jeol MSL 300 spectrometer. Gel permeation chromatography (GPC) was carried out with polystyrene standards in THF solutions. UV-Vis Spectrophotometer S-3100, of wavelength resolution of 0.95 nm, wavelength accuracy ±0.5 nm and wavelength reproducibility of ±0.02 nm. FT-IR spectrometer is of Bomem MB Series MB100. The Raman spectra were measured at room temperature using a confocal Raman microscope (WITec, Alpha 300 R) with a 632.8 nm He-Ne laser. In order to obtain the Raman spectra for the polymer and ILs mixtures, the incident laser beam was focused onto the sample using a microscope objective (100×). The scattered light was collected by same objective lens and dispersed by a grating and then detected by a charge-coupled-device array detector. Electrical conductivity measurements were performed using the standard four-in-line probe apparatus. And, the thickness of polymer thin films was measured with an Alpha-Step profilometer.

The structures of ILs and of polymer were optimized based on molecular mechanics and semi-empirical calculations using the HyperChem 7 molecular visualization and simulation program [56], [57]. Geometry optimizations based on molecular mechanics (using the MM+force field) and AM1 semi-empirical calculations were used to find the coordinates of molecular structures that represent a potential energy minimum. For geometry optimization using both molecular mechanics and semi-empirical calculations, the Polak-Ribiere routine with rms gradient of 0.01 as the termination condition was used. The minimum distance between solvent molecules and solute atoms was set at 2.3 Å. Molecular dynamics calculations were used to obtain a lower energy minimum by enabling molecules to cross potential barriers [56], [57]. The structures of ILs were optimized using semi-empirical calculations, and single-point calculations were carried out to determine the total energy and heat of formation.

#### Sample preparation

The 0.001 gm of polymer was dissolved in 3 ml of chloroform. Later, 1% wt of ILs was dissolved in this polymer chloroform mixture at room temperature and was then kept on vigorous stirring for 8 hrs. Further, the fully transparent solution was vacuum-dried at 40°C. We further continued with spectroscopic studies on this mixture.

## Results and Discussion

**Figure 1:**
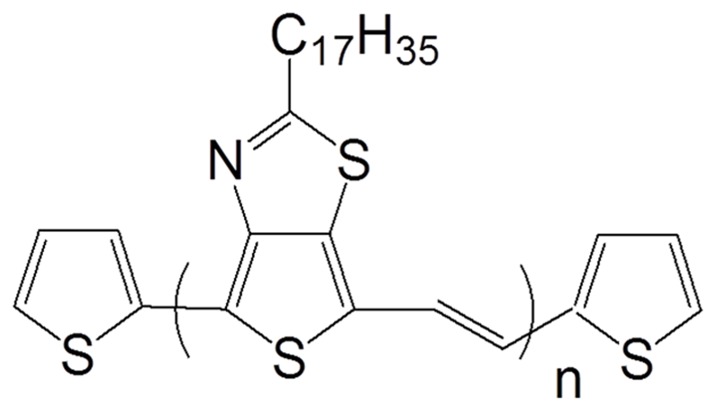
Schematically depiction of the low bandgap polymer structure (Poly(2-heptadecyl-4-vinylthieno[3,4-d]thiazole) (PHVTT)).

**Figure 2:**
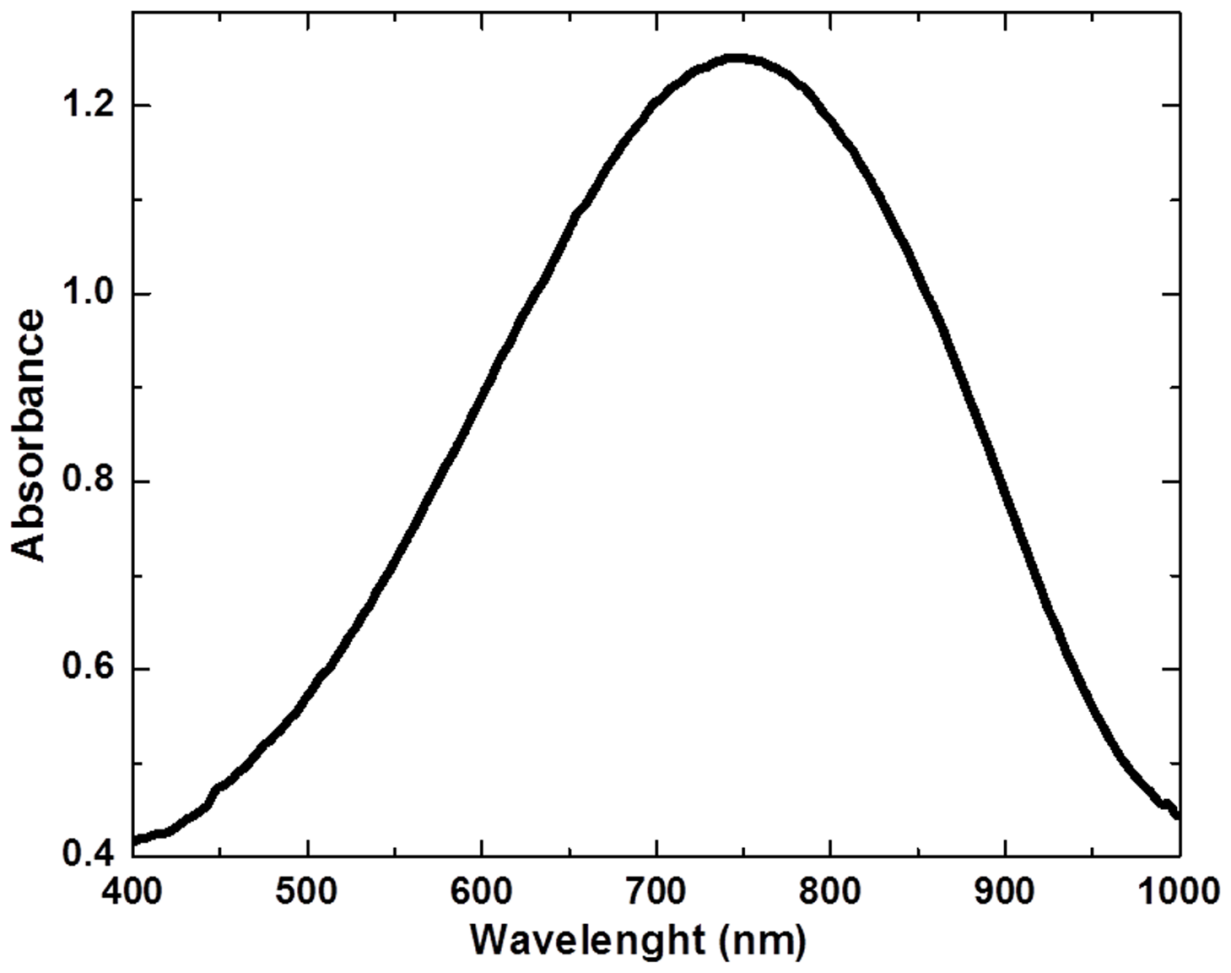
UV-visible absorption spectra of Poly(2-heptadecyl-4-vinylthieno[3,4-d]thiazole) (PHVTT) polymer.

The bandgap is very important for polymer based solar cells and there are several factors that influence the bandgap of polymer materials, such as intra-chain transfer, bond-length alternation, intermolecular interactions, aromaticity and π-conjugation length. Another major aspect of low bandgap polymer is the high energy level for HOMO of the donor and low energy level of for the LUMO of acceptor, that results in a low bandgap due to intra-chain charge transfer from donor to acceptor. After considering all these aspects, we demonstrated the design of a new low bandgap conjugated polymer, Poly(2-heptadecyl-4-vinylthieno[3,4-d]thiazole) (PHVTT). The structure of polymer is shown in Figure 1. UV-visible absorption spectra of polymer shows that the absorption onset of polymer is located at 750 nm, indicating an optical bandgap, as illustrated in Figure 2. Since the absorption band is beyond 600 nm, this clearly reveals that our polymer is a low bandgap polymer. As per literature, [41] this polymer will have the maximum harvest 35.6% of the available photons and giving rise to maximum theoretical current density 20.8 mA/cm^2^. As it was found that Poly(3-hexylthiophene) has absorption at 650 nm and thus it has the possibility to harvest only up to 22.4% of the available photons giving a maximum theoretical current density of 14.3 mA/cm^2^ [41]. In order to increase its efficiency, we have performed interaction studies of this synthesized low bandgap polymer (PHVTT) with ILs.

Combination of polymer and ILs as additives provides new progress, challenges and opportunities in polymer science. Therefore, we did interaction studies between the polymer and ILs using UV-Vis spectroscopy, Raman spectroscopy, FT-IR spectroscopy and semi-empirical calculations using the HyperChem 7. In addition, to this we have also studied the conductivity of polymer with or without ILs.

### Interaction Study of Polymer with ILs Using UV-Vis Spectroscopy

**Figure 3:**
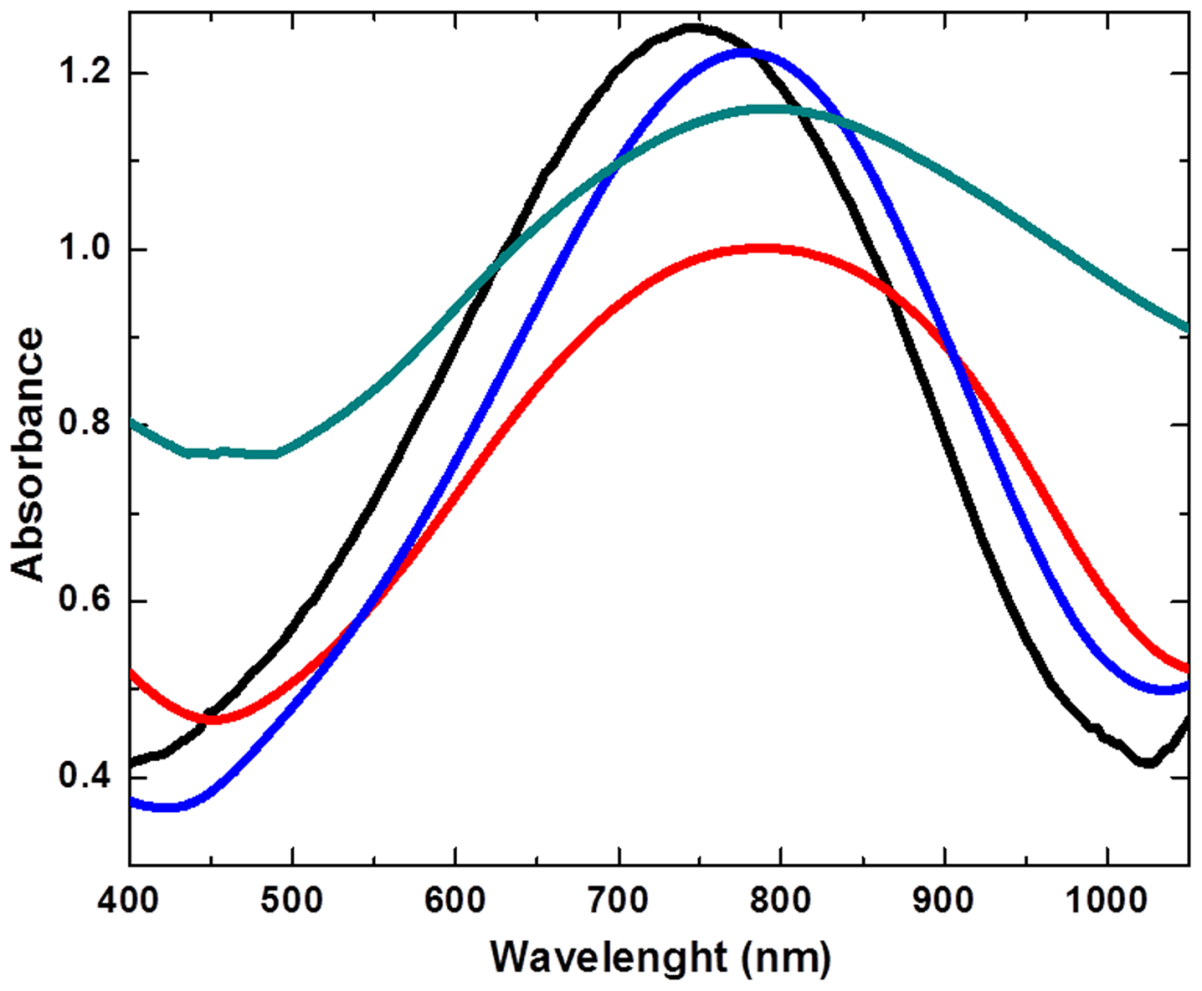
UV-visible absorption spectra of Poly(2-heptadecyl-4-vinylthieno[3,4-d]thiazole) (PHVTT) polymer (black) and polymer/ILs mixtures (Polymer+[N_1444_][MeSO4] (Red), Polymer+[Mim]Cl (blue) and Polymer+[Bmim]Cl (Dark cyan).

Miscibility between polymer and ILs is a quite important consideration for both mechanical and conductivity. And, the molecular interaction between polymer and ILs play an important role in controlling the miscibility factor. UV-Vis spectroscopy provides substantial experimental results of the molecular interaction between our synthesized polymer and ILs. All the ILs with polymer exhibited significant red shift observed as compared to polymer when studied alone, as displayed in Figure 3. The polymer showed the maximum absorbance at 750 nm, while the Poly(2-heptadecyl-4-vinylthieno[3,4-d]thiazole+[N_1444_][MeSO4] showed the maximum absorbance at 795 nm with quenching. This shows that the aprotic ammonium IL interact strongly with the low band gap polymer. On the other hand, the protic imidazolium ILs such as [Mim]Cl+polymer showed the red shift with maximum absorption to 781 nm with slight quenching. The slight change in intensity with red shift in [Mim]Cl+polymer reflects that the [Mim]Cl interacts with polymer without change in its original state. While the another aprotic imidazolium IL [Bmim]Cl when combined with the polymer also exhibited the red shift with 790 nm. Thus, UV-Vis spectroscopy results reveal that both protic and aprotic ILs interacts favorably with the polymer surface. Hence, all the studied ILs shows shift in the longer wavelength after the interaction with the polymer. Further, to support these interactions we made use of the confocal Raman spectroscopy.

### Interaction Study of Polymer with ILs Using Confocal Raman Spectroscopy

In order to explicate interactions we employed another reliable technique, confocal Raman spectroscopy that allowed us taking advantage of the spatial resolution of the confocal microscope [58]. Confocal Raman microscopy gives an important advantage of serving the vibrational energy of the target solutes as a reporter, which then eliminates the need for invasive monitoring aids such as molecular probes as is the case of confocal scanning laser microscopy [58]. Thus, we can collect the information on the relative concentration of a target solute at different depths inside a membrane polymer matrix, by simply moving the focalization spot across the membrane depth [59]. However, the Raman signal typically diminishes strongly with increasing penetration of the membrane polymer, owing to the Raman scattering as well as attenuation of the excitation laser power. Thus, the laser can be directed inside the polymer without damaging it, while maintaining a high selectivity and sensitivity.

**Figure 4:**
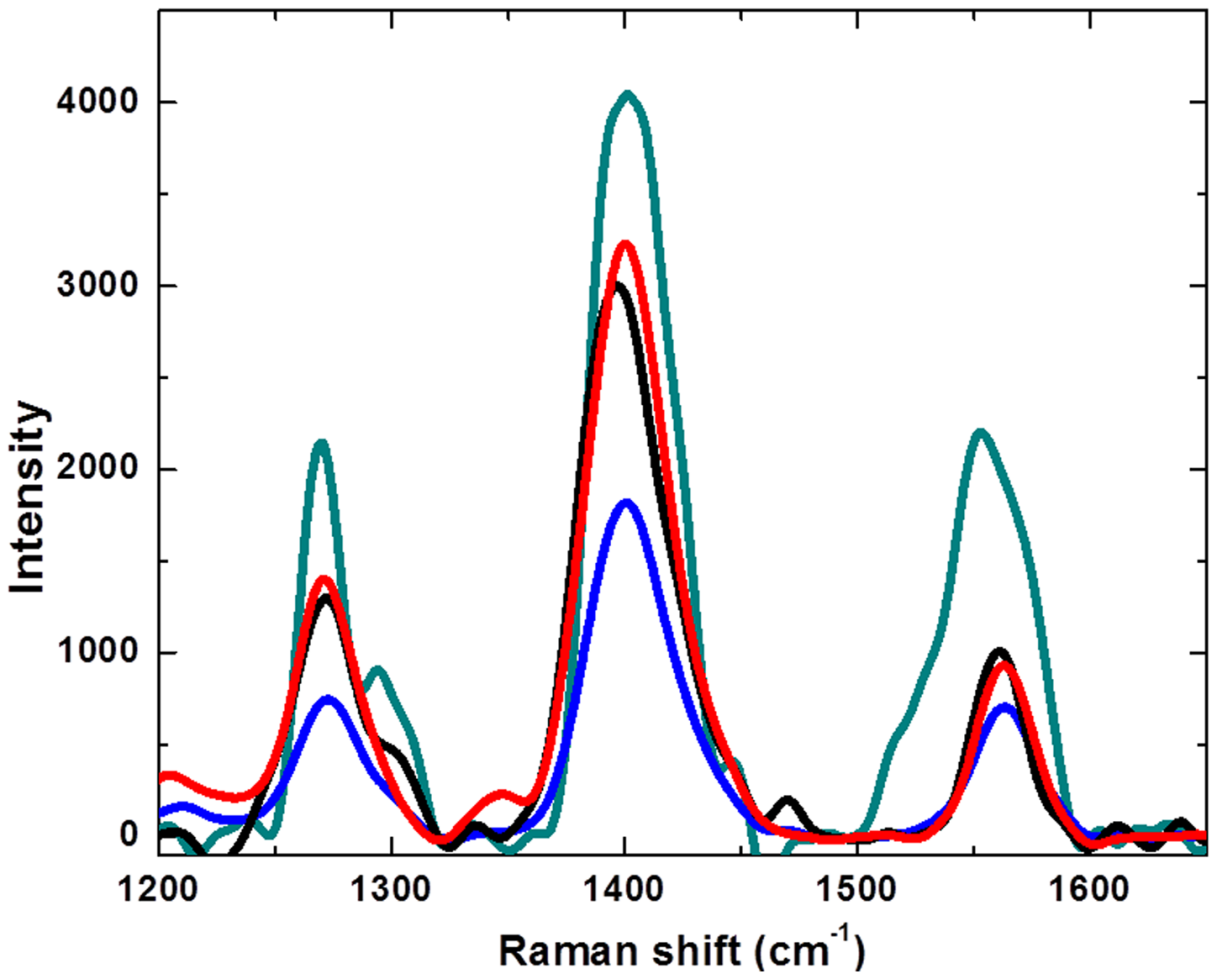
Confocal Raman spectra of Poly(2-heptadecyl-4-vinylthieno[3,4-d]thiazole) (PHVTT) polymer (black) and polymer/ILs mixtures (Polymer+[N_1444_][MeSO4] (Red), Polymer+[Mim]Cl (blue) and Polymer+[Bmim]Cl (Dark cyan).

### Interaction Study of Polymer with ILs Using FT-IR Spectroscopy and Theoretical Studies

The FT-IR spectroscopy and theoretical studies are other important tools for the study of interaction between the polymer and ILs. FT-IR spectra of low bandgap polymer in the absence and presence of various ILs are shown in Figure S1. Except thesome shifts in the spectra wavelength of polymer after the interaction with ILs, the polymer IR spectra are almost identical to the IR spectrum of polymer/ILs mixture. The band assignment is as follows: the absorption band 2920 and 2851 cm^−1^ are due to the alkyl chain attach to the polymer and 1521 and 1465 cm^−1^ band are due to the -C = C- bond of polymer. After the interaction of aprotic ammonium IL [N_1444_][MeSO4] with polymer, which showed the absorption band mainly due to alkyl chain shifts to longer wavelength at 2963 and 2876 cm^−1^. Also, the absorption due to -C = C- bond of polymer shifted to 1636 and 1470 cm^−1^. These shift in the absorption band revealed the interaction of polymer to the [N_1444_][MeSO4] IL. Although, we have also observed the FT-IR spectra of imidazolium ILs with polymer and we again found the shifts in the wavelength. In case of the protic imidazolium IL [Mim]Cl and polymer, we found the peaks at 2622, 2971, 1584 and 1551 cm^−1^ and for aprotic imidazolium IL [Bmim]Cl+polymer, peaks were observed at 2961, 2873, 1572 and 1465 cm^−1^. These bands associated with polymer and ILs clearly exposed the interaction between the two.

**Figure 5:**
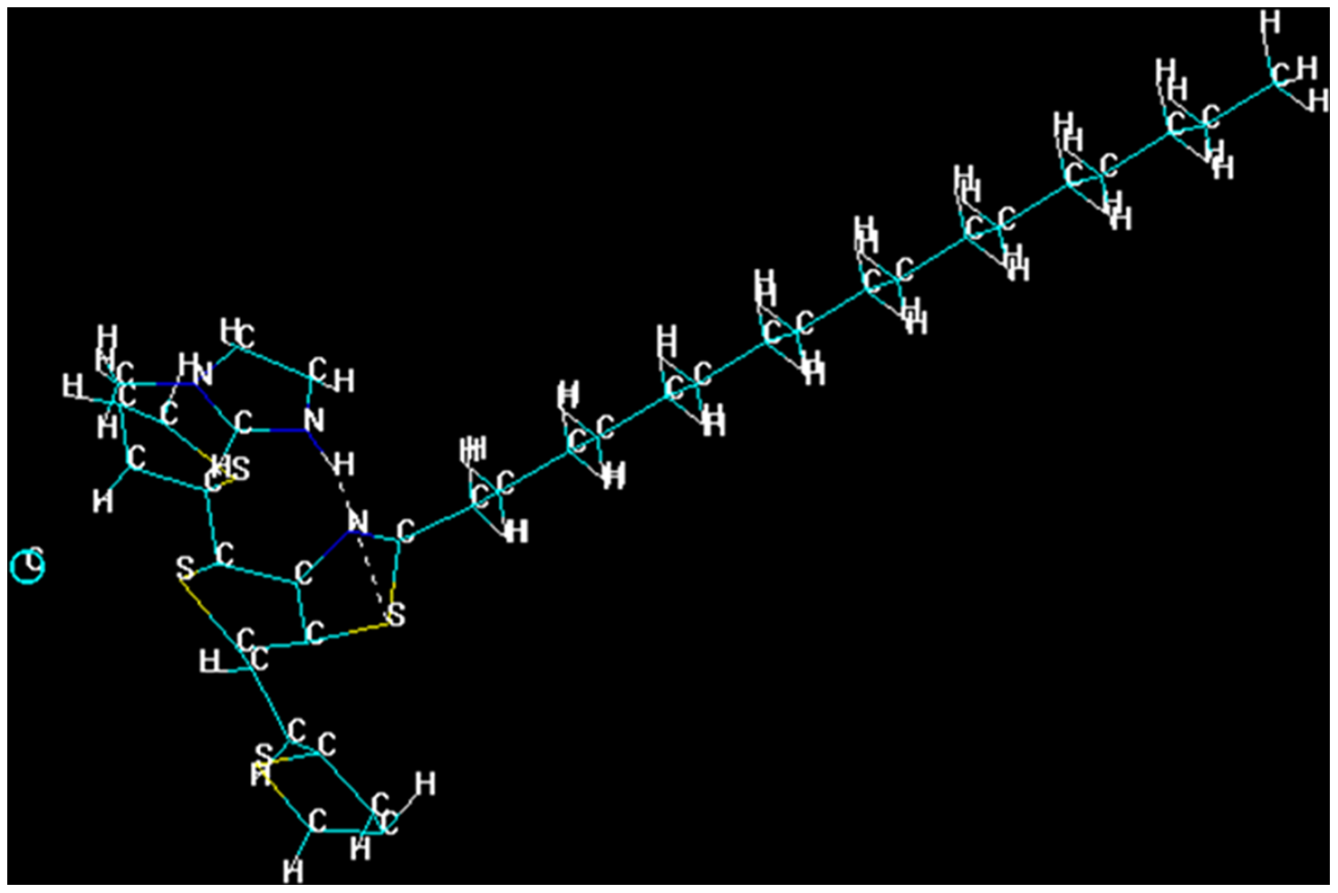
Hydrogen bonding interaction between polymer and [Mim]Cl molecules, which is predicted by semiempirical calculation with the help of Hyperchem 7.

The structures of ILs and polymer were optimized based on molecular mechanics and semi-empirical calculations using the HyperChem 7 molecular visualization and simulation program. Molecular model of polymer and ILs were constructed by the model builder of HyperChem. Initial molecular geometry of low bandgap polymer (Poly(2-heptadecyl-4-vinylthieno[3,4-d]thiazole) and ILs ([N_1444_][MeSO_4_], [Mim]Cl, and [Bmim]Cl) were optimized with the AM1 semi-empirical calculations, single point calculations were carried out to determine the total energies [57]. Now the optimized molecules, polymer (Poly(2-heptadecyl-4-vinylthieno[3,4-d]thiazole) and IL were chosen the placed on top of each other symmetrically (parallel) with a starting interplanar distance of 2.3 Å and the angle made by covalent bonds to the donor and acceptor atoms less than 120° was fulfilled. Further, the geometries were optimized using geometry optimizations based on molecular mechanics (using the MM+force field) and AM1 semi-empirical calculations, the Polak-Ribiere routine with rms gradient of 0.01 was used [57]. Hydrogen bonds were displayed using HyperChem “show hydrogen bonds” and “recompute hydrogen bond” options. Using this calculation, we examined that the protic imidazolium IL [Mim]Cl engaged in the H-bonding with the polymer as shown in Figure 5. Whereas, in other two aprotic ILs such as [N_1444_][MeSO4] and [Bmim]Cl, we noticed no H-bonding, but there might be ion pair or ion-dipole interaction which could lead to change in physio-chemical properties of polymer on the interaction with ILs, illustrated in Figures S2 and S3. These theoretical calculations are very well correlated with our experimental results, clearly affirming that there is nice interaction between the polymer and all studied ILs.

### Conductivity Study of Polymer with ILs

The enhancement in the conductivity of polymer is very important issue nowadays. There are many ways to increase the conductivity by adding secondary dopants or additives such as several alcohols (diethylene glycol, meso-erythritol 1,2,3,4,-tetrahydroxybutane, 2-nitroethanol, glycerol, or sorbitol) and high-boiling-point solvents (dimethyl sulfoxide, tetrahydrofurane, and dimethylformamide) [60]–[64]. Still there are many drawbacks in such system so we have tried to enhance the conductivity by making use of ILs in this piece of work. Our above experimental and theoretical data has shown that there is nice interaction between the polymer and ILs, hence there is quite possibility of enhancing the conductivity of polymer. And, our experimental conductivity results display in table 1, that polymer has conductivity of 7.0×10^−10^ S cm^−1^ at 25°C, whereas the addition of ILs boosts the conductivity of polymer. If we compare the conductivity of two aprotic ILs than the ammonium IL [N_1444_][MeSO4]+polymer, this mixture has conductivity of 4.0×10^−7^ S cm^−1^. Whereas, conductivity of another aprotic IL [Bmim]Cl+polymer is 2.7×10^−6^ S cm^-1^, which is quite higher than aprotic ammonium IL. On the other hand, the protic IL [Mim]Cl+polymer has very high conductivity with polymer of about 2.6×10^−5^ S cm^−1^.

**Table 1:** Conductivity of polymer and polymer/ILs mixtures.

Sample	Conductivity (Scm^−1^)
Polymer	7.0×10^−10^
Polymer+[N_1444_][MeSO4]	4.0×10^−7^
Polymer+[Mim]Cl	2.6×10^−5^
Polymer+[Bmim]Cl	2.7×10^−6^

It is difficult to draw conclusion for the difference in conductivity on the basis of the structure of ILs. But there might be a possibility that the H-bonding between protic IL and polymer increases its conductivity to greater extent than other ILs. This relationship can further be interpreted as a combination of the H-bonding and electron-withdrawing properties of the polymer. The pi electrons are apparently involved in the generation of charge carriers, and any substituent which tends to reduce the electron density of the system produces a rather substantial increase in activation energy (*E_a_).* In competition to this effect, is the decrease in *E_a_* produced by extended intermolecular overlap which in turn is caused by the H-bonding. In another words, this might be due to the increase in number of electrons involved in H-bond, which contribute to enhancement in conductivity as compared to those system where H-bonding is absent.

To conclude, our extensive experimental data illustrated that ammonium and imidazolium based ILs interacts strongly with newly synthesized low bandgap polymer. Further, to obtain some insight into interaction between polymer and IL, we have exploited the UV-Vis spectroscopy, Raman spectroscopy, FT-IR spectroscopy and semi-empirical calculations using the HyperChem 7. We examined that protic imidazolium IL has a greater tendency to form hydrogen bonds as compared to aprotic ILs. These observed interactions are well supported by our theoretical calculations, which are obtained by Hyperchem 7. Further, all ILs showed very good performance as conductivity enhancer, while the protic IL showed the highest conductivity. These results are very useful in the field of low bandgap polymer and can also help in increasing the performance of optical devices.

## Supporting Information

Figure S1 FT-IR spectra of (a) Polymer ((Poly(2-heptadecyl-4-vinylthieno[3,4-d]thiazole) (PHVTT)), (b) Polymer+[Bmim]Cl, (c) Polymer+[Mim]Cl and Polymer+[N_1444_][MeSO_4_].
(TIF)

Figure S2 Molecular interaction between polymer and [N_1444_][MeSO_4_]molecules, which is predicted by semiempirical calculation with the help of Hyperchem 7.
(TIF)

Figure S3 Molecular interaction between polymer and [Bmim]Cl molecules, which is predicted by semiempirical calculation with the help of Hyperchem 7.
(TIF)
