# Supplementary figures and images for: Elucidating Interactions and Conductivity of Newly Synthesised Low Bandgap Polymer with Protic and Aprotic Ionic Liquids
Source: PLoS One. 2013 Jul 9;8(7):e68970. doi: 10.1371/journal.pone.0068970 (PMC3706474; doi:10.1371/journal.pone.0068970)

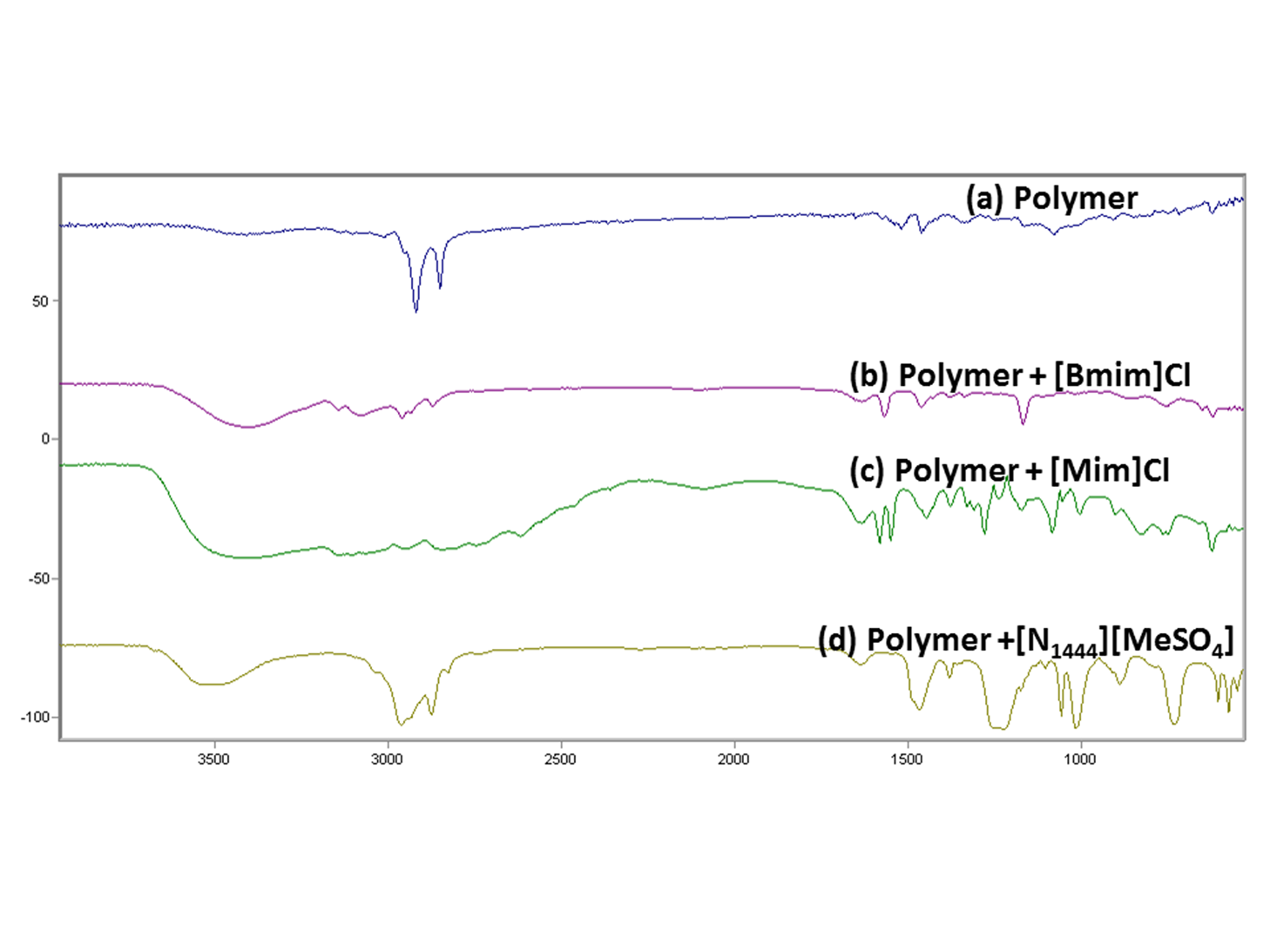

Supplement: Figure S1 — FT-IR spectra of (a) Polymer ((Poly(2-heptadecyl-4-vinylthieno[3,4-d]thiazole) (PHVTT)), (b) Polymer+[Bmim]Cl, (c) Polymer+[Mim]Cl and Polymer+[N1444][MeSO4]. (TIF) [file pone.0068970.s001.tif]

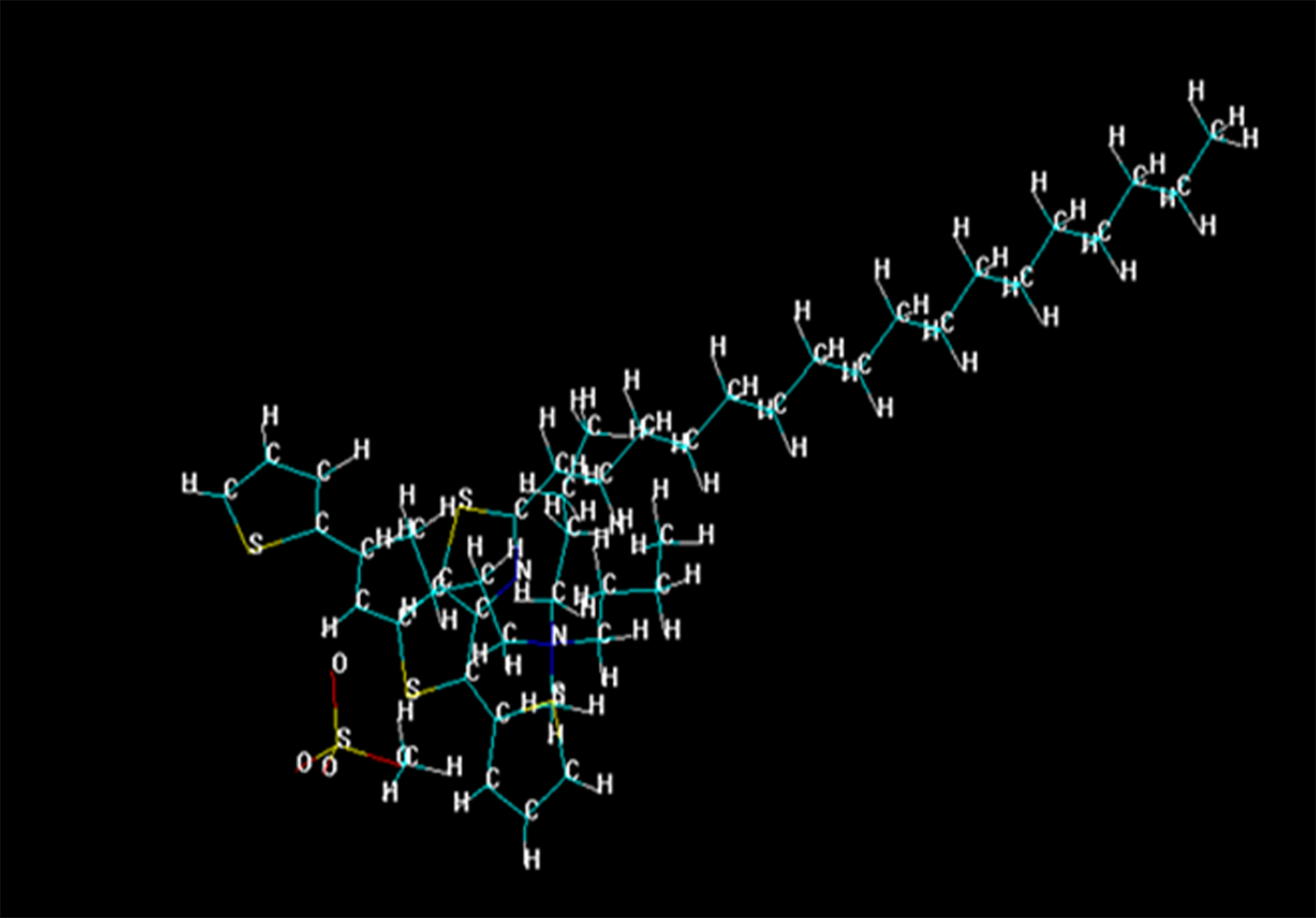

Supplement: Figure S2 — Molecular interaction between polymer and [N1444][MeSO4]molecules, which is predicted by semiempirical calculation with the help of Hyperchem 7. (TIF) [file pone.0068970.s002.tif]

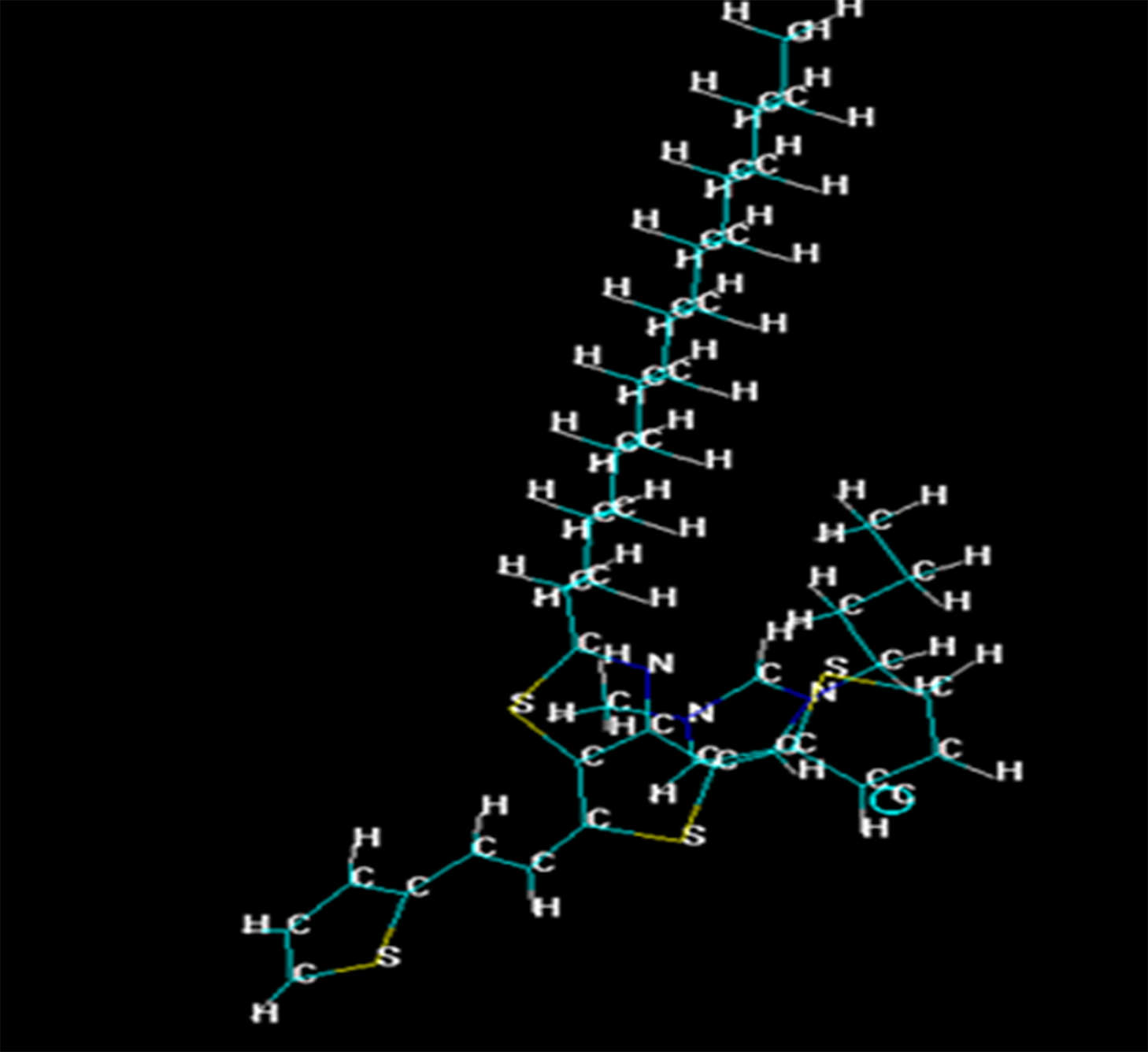

Supplement: Figure S3 — Molecular interaction between polymer and [Bmim]Cl molecules, which is predicted by semiempirical calculation with the help of Hyperchem 7. (TIF) [file pone.0068970.s003.tif]
